# Pregnancy, Mental Well-Being and Lockdown: A Nationwide Online Survey in France

**DOI:** 10.3390/healthcare10101855

**Published:** 2022-09-23

**Authors:** Laurent Gaucher, Chloé Barasinski, Corinne Dupont, Chantal Razurel, Swann Pichon, Emma Leavy, Sylvie Viaux-Savelon, Marion Cortet, Nicolas Franck, Frédéric Haesebaert, Julie Haesebaert

**Affiliations:** 1 Midwifery Department, Geneva School of Health Sciences, HES-SO University of Applied Sciences and Arts Western Switzerland, CH-1206 Geneva, Switzerland; 2 Research on Healthcare Performance (RESHAPE) INSERM U1290, Pôle de Santé Publique, Hospices Civils de Lyon, Université Claude Bernard Lyon 1, F-69008 Lyon, France; 3 Institut Pascal Axe TGI-DecisiPH. CNRS, Clermont Auvergne INP, CHU Clermont-Ferrand, Université Clermont Auvergne, F-63000 Clermont-Ferrand, France; 4 Réseau Périnatal Aurore, F-69004 Lyon, France; 5 Hospices Civils de Lyon, Hôpital de la Croix-Rousse, F-69004 Lyon, France; 6 Centre Ressource de Réhabilitation Psychosociale et de Remédiation Cognitive (Ressource Center for Psychosocial Rehabilitation and Cognitive Remediation), Hôpital Le Vinatier, F-69500 Bron, France; 7 UMR, 5229, CNRS & Université Lyon 1, Université de Lyon, F-69008 Lyon, France; 8 Centre Référent Lyonnais de Réhabilitation Psychosociale CL3R, Centre Hospitalier Le Vinatier, F-69500 Bron, France; 9 INSERM U1028, CNRS UMR5292, PSYR2 Team, Lyon Neuroscience Research Center, Université Claude Bernard Lyon 1, F-69500 Bron, France

**Keywords:** pregnant women, quarantine, mental health, pandemic, survey

## Abstract

The objective of this study was to compare the mental well-being of French women who were and were not pregnant during the first COVID-19 pandemic lockdown. We performed a nationwide online quantitative survey including all women between 18 and 45 years of age during the second and third weeks of global lockdown (25 March–7 April 2020). The main outcome measure was mental well-being measured by the Warwick–Edinburgh Mental Well-Being Scale (WEMWBS). This study analysed 275 responses from pregnant women and compared them with those from a propensity score–matched sample of 825 non-pregnant women. In this French sample, the median WEMWBS score was 49.0 and did not differ by pregnancy status. Women living in urban areas reported better well-being, while those with sleep disorders or who spent more than an hour a day watching the news reported poorer well-being. During the first lockdown in France, women had relatively low mental well-being scores, with no significant difference between pregnant and non-pregnant women. More than ever, health-care workers need to find a way to maintain their support for women’s well-being. Minor daily annoyances of pregnancy, such as insomnia, should not be trivialised because they are a potential sign of poor well-being.

## 1. Introduction

In some Western countries, suicides are one of the main causes of maternal deaths [1,2]. Long before the COVID-19 pandemic, a systematic review found that between 7 and 13% of women are depressed during pregnancy and 19% have postpartum depression; 7% of these cases were considered major [3]. We also know that the mental disorders of mothers are strongly associated with their children’s physical and mental well-being [4].

At the beginning of the COVID-19 pandemic period, the medical situation was considered much more anxiety inducing for pregnant than for non-pregnant women. In March 2020, no data were available about the potential for a higher risk of severe effects due to this coronavirus during pregnancy, for both mother and child, by possible vertical transmission [5]. Applying the precautionary principle, pregnant women were considered to be at high risk of medical complications [6]. The separation of an infected mother from the child at birth was debated [7] and many French hospitals prevented women (infected or uninfected) from receiving support from their partners during childbirth [8]. The lockdown measures, imposed to limit the epidemic’s spread and applied to maternity wards in elsewhere in Europe and in Western countries, have raised concerns among professionals about their psychological impact on pregnant women and mothers [9]. The reorganisation of hospitals and the community care sector may have generated concern about access to care during pregnancy and childbirth [10].

These factors indicate that the current pandemic period, with its repeated lockdowns, is likely to negatively affect the mental well-being of pregnant women [11,12]. Most of the recently reviewed studies have reported that isolation has negative psychological effects on the population, including anxiety, depression, and post-traumatic and other stress symptoms [13,14]. While some controlled comparative studies on the lockdown’s impact on depression during the postpartum period are available, to our knowledge, no such data exist for broader outcomes such as mental well-being among pregnant women in Western countries [15,16,17].

We therefore sought to compare the mental well-being level of French pregnant and non-pregnant women during the first COVID-19 lockdown. As a secondary objective, we examined the association between pregnant women’s characteristics and their level of well-being.

## 2. Materials and Methods

We conducted a nationwide online survey to measure the mental well-being of French women during the second and third weeks of global lockdown during the pandemic. The results of this quantitative study are reported according to the Checklist for Reporting Results of Internet E-Surveys (cf. Table S1: CHERRIES) [18].

### 2.1. Screening and Recruitment

The LockUwell survey was an open French e-survey. Recruitment took place by sending the survey link through various online announcements on social networks (Facebook, Twitter and LinkedIn, the authors’ individual and institutional accounts) and national newspaper websites. It directed those interested in participating to this survey, created with open-source software (LimeSurvey). Individual consent was obtained from all women. In accordance with current French legislation on health research, no ethics committee approval was required because data collection was anonymous. We obtained a convenience sample through voluntary participation, without any incentives or rewards. The survey was open throughout the first nationwide lockdown period. The analysis presented here studies data collected from 25 March to 7 April (week 2 and week 3 of this first national lockdown in France). We used cookies to ensure we collected only one set of answers per participant.

The LockUwell survey targeted all French-speakers. This analysis includes pregnant and non-pregnant women aged between 18 and 45 years. We thus excluded all men, women older or younger than the selected age group, as well as women locked down outside France and those who did not know their pregnancy status. The completion rate was defined by the ratio of users who finished the survey divided by the number who agreed to participate.

### 2.2. Measures and Definitions

The questionnaire of the LockUwell survey was constructed through an iterative testing process that included revisions by epidemiologists, psychiatrists in several subspecialties, mental-health service users, and citizens, as described elsewhere [19]. The survey included sociodemographic data (Section 1), an evaluation of well-being (validated French version of the Warwick–Edinburgh Mental Well-Being Scale, WEMWBS) (Section 2), stress evaluations (Section 3), medical, psychiatric, lockdown and isolation, and social contact history (Section 4), personal situation (infection or exposure of self or family, friends, and co-workers) regarding COVID-19 (Section 5), as well as personal and environmental conditions during lockdown including watching news, physical exercise and sports activities, and sleep disorders (Section 6) [20,21]. The items were not randomised. Respondents were able to review and change their answers through a back button. The estimated duration of the questionnaire was 15 to 30 min.

### 2.3. Statistical Analysis

All statistical analyses were performed with R software, version 4.0.3 [22]. Inescapably, if only for their age, non-pregnant women who responded to our survey did not have the same characteristics as the pregnant women who responded to it. Therefore, we used a propensity score approach to control for confounding factors that might influence our result on their mental well-being levels. We included all pregnant women but selected non-pregnant women by stratification by a propensity score [23]. We analysed only questionnaires with sufficient information to calculate this score. A woman’s propensity score was defined as her probability of being pregnant based on the individual covariates we measured. This score was calculated by applying a generalised linear model with current pregnancy as the dependent variable and considering the following characteristics: age range, marital status, living alone or with someone else, psychiatric (including addictions) history, parity, local extent of pandemic area during weeks 2–3 of lockdown, educational level, and occupation. We distributed the propensity scores obtained for each woman into five classes. Finally, we matched non-pregnant women (controls) on a three-for-one basis, class by class. The early/late pandemic area was determined retrospectively as early or late by the respondents’ postcodes. Districts with a ratio of more than 2 deaths per 100,000 residents on 23 March 2020, were classified as early pandemic areas by the French national public health agency, Santé Publique France (https://www.data.gouv.fr/ accessed on 5 September 2021).

Quantitative variables with normal distributions according to the Shapiro–Wilk test were described by their means and standard deviations (SD), and then compared with a Welch two-sample t-test. When distributions were not normal, variables were described according to their medians, with their 25th and 75th percentiles (Q1–Q3), and then compared by a Wilcoxon rank sum test. Qualitative variables were described as the number of individuals and percentages and then compared with Fisher’s exact test. The denominator is reported when it comprises less than 95% of the total sample size. A multiple regression analysis then assessed the association of pregnant women’s characteristics with their WEMWBS score. This score was entered as a dependent variable in the model and all their other characteristics as independent variables.

## 3. Results

**Figure 1:**
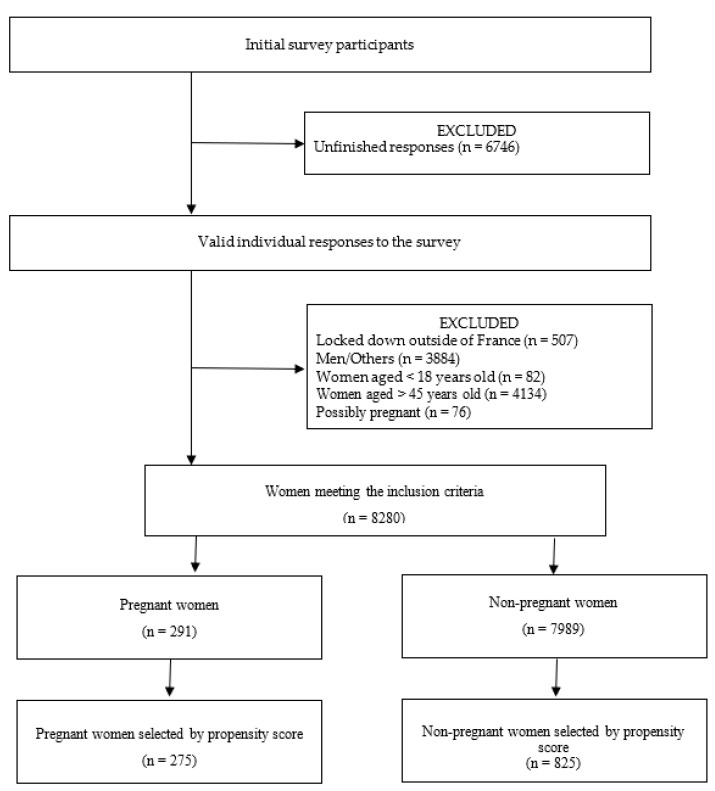
Study flow.

Of the 23,709 questionnaires started, only 16,963 provided sufficient responses for analysis, for a completion rate of 71.55%. Of these 16,963 participants, 291 women were pregnant, a figure consistent with the around 800 000 annual births in France for a population of 67 million persons. After exclusion of women without minimal data to calculate the propensity score, 275 pregnant women and 825 non-pregnant women were included and analysed (Figure 1). These pregnant women had a mean age of 31 years (SD = 4.1), and 97% were in a relationship. Most were nulliparous (59%), with a high educational level (63%) and no history of psychiatric disorder, including addiction (83%) (Table 1). These pregnant women had a median WEMWBS score of 49 (Q1–Q3 43.0–54.0), as did the non-pregnant women (Q1–Q3 44.0–54.0) (*p* = 0.720).

Among pregnant women, suburban living was significantly associated with a lower level of well-being (47 vs. 50 for urban living). Sleep disorders were similarly significantly associated with poorer well-being (45.5 WEMBWS median), as was watching the news for more than an hour a day (47 WEMBWS median; Table 2). Pregnant women who were in relationships, or had a high level of education, or who worked alternately at home and at the office, tended to report higher levels of mental well-being, although these differences were not statistically significant.

## 4. Discussion

### 4.1. Main Findings

The COVID-19 lockdown appeared to affect the well-being of pregnant and non-pregnant women equally. Our study identified important characteristics of pregnant women that appears to be associated with poorer mental well-being. These included suburban residence, sleep disorders, and spending more than one hour a day watching the news. Midwives should explore these warning signs.

### 4.2. Strengths and Limitations

The originality of this study is its approach based on women’s well-being scores. Well-being is a key determinant of health-related behaviours [24]. The scale used in our study combines the hedonic approach (positive emotions, satisfaction with one’s life) with the eudemonic approach that includes the perception of usefulness and confidence in the future, which may be particularly questionable in the current context of media gloom-mongering. Most of the other tools published so far have assessed negative psychological reactions such as anxiety and stress, or even pathological reactions such as depression and/or post-traumatic stress disorder. The main strengths of our study are that the results are based on a voluntary general population survey with the control group selected by a propensity score as a representative sample of our source sample. Thus, this study is based on a convenience sample with overrepresentation of high educational and socioeconomic levels with stable partner situations, who are at lower risk of stress. A noteworthy limitation of our study is due to the selection bias inherent in any population-based survey [25]. Indeed, on the one hand, our study was only accessible to women with access to the internet and, on the other hand, our study shows that access to information (especially available on the internet) was associated with the level of well-being. Nevertheless, one the one hand, more than 98% of the French women from which our sample emerged have access to the internet [26]. One the other hand, our method balances the selection bias between the groups and therefore provides some confidence in our results regarding the main objective of comparing well-being according to pregnancy [27].

### 4.3. Interpretation

Given that previous studies have reported similar levels of well-being between men and women, pregnant or not, we hypothesised that the specific official measures affecting pregnant women compared with non-pregnant women based on the precautionary principle might have compromised their well-being [21,28]. We were surprised that we did not observe any difference in well-being between pregnant and non-pregnant women. This may be explained in part by the fact that work is also a major source of stress, from which some pregnant women are protected [29]. This hypothesis was also suggested to explain the decrease in the preterm birth rate during the lockdown [30]. During the French lockdown, many pregnant women, especially among those working as caregivers, were declared temporarily unavailable for work to protect them. Our study showed a lower level of well-being among women in general and pregnant women in particular during than before lockdown: 49 for the WEMSBS total score for both groups during lockdown in our study vs. 53 among the French general population in 2014 (not of women, but identical in the one group primarily female, and the one primarily male) [21] and 54 among British women pregnant with their first child in 2016–2017 [28]. These results are in line with those recently published about a population recruited in the United States, the United Kingdom and Ireland [12]. However, our results should be seen in the French context. Indeed, there are large cultural and policy differences between European countries that are reflected, for example, in large differences in sick leave rates [31]. Lastly, let us consider that, apart from the specific restrictions for pregnant women, the main restriction of confinement that applied to all women may have been particularly burdensome for non-pregnant mothers who were working at home while caring for their child(ren) (more so than for pregnant women without children). A recent Irish study also shows a lower perceived level of social support among pregnant women [32]. The lack of a significant association between the presence of psychiatric history or addiction and well-being could be related to a selection bias in our sample but suggests the importance of caring for the well-being of all pregnant women, regardless of their history. Contrary to results from a Chinese study, living in urban-based environments appears to be a protective factor for well-being [33]. Recent studies have showed a negative impact of rural living on mental health in Turkey and Italy [34,35]. However, fear of contracting the virus and being locked down are legitimate factors that may affect women’s well-being.

### 4.4. Research Recommendation

Further studies should be conducted to assess the impact of repeated lockdowns on pregnant women, and indeed on mothers, especially those who work. We expect that further research with a similar methodology and better control of selection bias will confirm our results. It will also be important to study women’s well-being by designs appropriate for recruiting residents with low literacy levels. Last but not least, this pandemic seems to have led to the population losing confidence in the future, resulting in a fall in the birth rate. The increase that followed remained well below the rates of previous years [36].

### 4.5. Practical Recommendation

With the pandemic still active as we go from lockdown to lockdown, the first implication for clinical practice is the importance of maintaining contact with pregnant women, especially those in suburban areas. New ways must be found to maintain this supportive contact. The postponement or cancellation of consultations deemed non-essential by midwives has limited the support available to women during lockdown [10,37]. Remote video consultation is an innovative approach that has already shown its effectiveness in reducing antenatal distress and pregnancy-related anxiety; it also raises questions in terms of accessibility and literacy [38]. A second implication for clinical practice is that midwives should be especially observant of women’s sleep disorders. A Finnish study showed that although the lockdown was not associated with total sleep time, daily rhythms changed, and pregnant women overall fell asleep later and woke up later [39]. Another study found a correlation between COVID-related stress and sleep disturbances [40]. Although sleep disorders are common during pregnancy, they must never be considered insignificant. They constantly affect women’s well-being and quality of life [41,42]. Sleep psychoeducation is another approach to helping these women [43].

Finally, we can suggest that might be useful for health-care workers, especially for midwives, to communicate clearly and visibly with women about the impact of the pandemic on pregnancy to counterbalance the negative effect of the media.

## 5. Conclusions

In this survey, the level of mental well-being of pregnant women was similar to that of non-pregnant women during the first lockdown. More than ever, clinicians need to find a way to maintain support for women’s well-being and to screen for potential symptoms of mental distress.

## Figures and Tables

**Table 1:** Characteristics of study participants by pregnancy status.

		Pregnant (n = 275)	Non-Pregnant (n = 825)	*p*
Age in years, n (%)	[18–25]	13 (4.7)	33 (4.0)	0.809
	[26–35]	219 (79.6)	670 (81.2)	
	[36–45]	43 (15.6)	122 (14.8)	
Marital status, n (%)	Single	7 (2.5)	29 (3.5)	0.736
	Divorced or widowed	1 (0.4)	3 (0.4)	
	In a relationship	267 (97.1)	793 (96.1)	
No. of people in household, n (%)	1	18 (6.5)	57 (6.9)	0.945
	≥2	257 (93.5)	768 (93.1)	
Psychiatric (including addiction) history, n (%)	Ongoing	16 (5.8)	86 (10.4)	0.065
	Past	30 (10.9)	77 (9.3)	
	No history	229 (83.3)	662 (80.2)	
Pandemic area, n (%)	High-risk	144 (52.4)	438 (53.1)	0.889
	Low-risk	131 (47.6)	387 (46.9)	
Parity, n (%)	Nulliparous	161 (58.5)	462 (56.0)	0.505
	Parous	114 (41.5)	363 (44.0)	
Educational level (ISCED 2011) *, n (%)	≤3	21 (7.6)	78 (9.5)	0.603
	4–6	80 (29.1)	246 (29.8)	
	≥7	174 (63.3)	501 (60.7)	
WEMWBS total score (from 14 to 70)	Median (25–75th pctl)	49.0 (43.0–54.0)	49.0 (44.0–54.0)	0.720
Work-related stress (from 0 to 10) **	Median (25–75th pctl)	5 (3–7)	6 (3–7)	0.141
Personal stress (from 0 to 10)	Median (25–75th pctl)	5 (3–7)	5 (3–7)	0.845
Overall stress (from 0 to 10)	Median (25–75th pctl)	5 (3–7)	5 (4–7)	0.117
Watches news > 1 h/day, n (%)		76 (26.6)	207 (25.1)	0.449
Sports/exercise > 30 min daily, n (%)		69 (25.1)	354 (42.9)	<0.001
Sleep disorder, n (%)		70 (25.5)	210 (25.5)	>0.99

* ISCED level 3: Upper Secondary (high school). ISCED level 7: Master’s Degree. ** Excluding unemployed women (118 pregnant and 446 non-pregnant women). WEMWBS: Warwick–Edinburgh Mental Well-Being Scale.

**Table 2:** Linear regression for total WEMWBS scores of pregnant women (n = 275).

		WEMWBS Scores			
Predictors		Median	Estimated β	CI	*p*
Age in years	[18–25]	52	Reference (0.00)		
	[26–35]	48	−2.78	−6.77 to 1.22	0.172
	[36–45]	49	−2.08	−6.65 to 2.50	0.372
Marital status	In a relationship	49	Reference (0.00)		
	Divorced, or widowed	41	−3.03	−16.90 to 10.85	0.668
	Single	42	−4.09	−10.04 to 1.86	0.177
Working during lockdown	At the workplace	46.5	Reference (0.00)		
	Telecommuting	51	2.79	−3.02 to 8.60	0.345
	Mixed	51.5	5.19	−2.21 to 12.58	0.168
	Unemployed	48	1.71	−4.09 to 7.51	0.562
Educational level (ISCED 2011)	≤3	44	Reference (0.00)		
	4–6	48	0.64	−2.71 to 3.98	0.709
	≥7	50	2.24	−1.17 to 5.65	0.196
Psychiatric (including addiction) history	No history	49	Reference (0.00)		
	Past	48	−0.17	−2.91 to 2.58	0.905
	Ongoing	45	−0.74	−4.48 to 3.00	0.698
Outdoor space	No	46.5	Reference (0.00)		
	Yes	49	2.52	−0.01 to 5.04	0.051
Housing location	Urban	50	Reference (0.00)		
	Suburban	47	−2.41	−4.52 to −0.29	**0.026**
	Rural	48.5	−0.70	−2.85 to 1.45	0.521
Living alone	No	49	Reference (0.00)		
	Yes	44.5	−1.98	−5.84 to 1.87	0.313
Parity	Nulliparous	50	Reference (0.00)		
	Parous	48	−0.26	−2.05 to 1.53	0.775
Pregnancy period	First and second trimester	49	Reference (0.00)		
	Last three months	48	−0.17	−2.05 to 1.71	0.858
Watching news	≤1 h	50	Reference (0.00)		
	>1 h	47	−2.08	−3.93 to −0.23	**0.028**
Sports/exercise	≤30 min daily	48	Reference (0.00)		
	>30 min daily	51	1.05	−0.92 to 3.03	0.294
Sleep disorder	No	50	Reference (0.00)		
	Yes	45.5	−2.99	−4.88 to −1.10	**0.002**

## Data Availability

The data that support the findings of this study are available from the corresponding author upon reasonable request.

## Supplementary Materials

The following supporting information can be downloaded at: https://www.mdpi.com/article/10.3390/healthcare10101855/s1, Table S1: Checklist for Reporting Results of Internet E-Surveys (CHERRIES).

## References

[1] Deneux-Tharaux C., Saucedo M. (2021). Les Morts Maternelles en France: Mieux Comprendre Pour Mieux Prévenir. 6e Rapport de l’Enquête Nationale Confidentielle sur Les Morts Maternelles (ENCMM) 2013–2015.

[2] Oates M. (2003). Perinatal Psychiatric Disorders: A Leading Cause of Maternal Morbidity and Mortality. Br. Med. Bull..

[3] Gavin N.I., Gaynes B.N., Lohr K.N., Meltzer-Brody S., Gartlehner G., Swinson T. (2005). Perinatal Depression: A Systematic Review of Prevalence and Incidence. Obstet. Gynecol..

[4] Stein A., Pearson R.M., Goodman S.H., Rapa E., Rahman A., McCallum M., Howard L.M., Pariante C.M. (2014). Effects of Perinatal Mental Disorders on the Fetus and Child. Lancet.

[5] Chen H., Guo J., Wang C., Luo F., Yu X., Zhang W., Li J., Zhao D., Xu D., Gong Q. (2020). Clinical Characteristics and Intrauterine Vertical Transmission Potential of COVID-19 Infection in Nine Pregnant Women: A Retrospective Review of Medical Records. Lancet.

[6] Favre G., Pomar L., Qi X., Nielsen-Saines K., Musso D., Baud D. (2020). Guidelines for Pregnant Women with Suspected SARS-CoV-2 Infection. Lancet Infect. Dis..

[7] (2020). Coronavirus (COVID-19) Infection in Pregnancy. Information for Healthcare Professionals; The Royal College of Midweves, Royal College of Obstetrcians & Gynaecologists. https://www.rcog.org.uk/media/xsubnsma/2022-03-07-coronavirus-covid-19-infection-in-pregnancy-v15.pdf.

[8] Peyronnet V., Sibiude J., Deruelle P., Huissoud C., Lescure X., Lucet J.-C., Mandelbrot L., Nisand I., Vayssière C., Yazpandanah Y. (2020). Infection par le SARS-CoV-2 chez les femmes enceintes: État des connaissances et proposition de prise en charge par CNGOF. Gynécologie Obs. Fertil Sénologie.

[9] Viaux S., Maurice P., Cohen D., Jouannic J. (2020). Giving Birth under Lockdown during the COVID-19 Epidemic. J. Gynecol. Obstet. Hum. Reprod..

[10] Baumann S., Gaucher L., Bourgueil Y., Saint-Lary O., Gautier S., Rousseau A. (2021). Adaptation of Independent Midwives to the COVID-19 Pandemic: A National Descriptive Survey. Midwifery.

[11] Huang Y., Zhao N. (2020). Generalized Anxiety Disorder, Depressive Symptoms and Sleep Quality during COVID-19 Epidemic in China: A Web-Based Cross-Sectional Survey. Psychiatry Res..

[12] Pope J., Olander E.K., Leitao S., Meaney S., Matvienko-Sikar K. (2021). Prenatal Stress, Health, and Health Behaviours during the COVID-19 Pandemic: An International Survey. Women Birth J. Aust. Coll. Midwives.

[13] Brooks S.K., Webster R.K., Smith L.E., Woodland L., Wessely S., Greenberg N., Rubin G.J. (2020). The Psychological Impact of Quarantine and How to Reduce It: Rapid Review of the Evidence. Lancet.

[14] Odriozola-González P., Planchuelo-Gómez Á., Irurtia M.J., de Luis-García R. (2020). Psychological Effects of the COVID-19 Outbreak and Lockdown among Students and Workers of a Spanish University. Psychiatry Res..

[15] Zanardo V., Manghina V., Giliberti L., Vettore M., Severino L., Straface G. (2020). Psychological Impact of COVID-19 Quarantine Measures in Northeastern Italy on Mothers in the Immediate Postpartum Period. Int. J. Gynaecol. Obstet. Off. Organ Int. Fed. Gynaecol. Obstet..

[16] Molgora S., Accordini M. (2020). Motherhood in the Time of Coronavirus: The Impact of the Pandemic Emergency on Expectant and Postpartum Women’s Psychological Well-Being. Front. Psychol..

[17] Oskovi-Kaplan Z.A., Buyuk G.N., Ozgu-Erdinc A.S., Keskin H.L., Ozbas A., Moraloglu Tekin O. (2020). The Effect of COVID-19 Pandemic and Social Restrictions on Depression Rates and Maternal Attachment in Immediate Postpartum Women: A Preliminary Study. Psychiatr. Q..

[18] Eysenbach G. (2004). Improving the Quality of Web Surveys: The Checklist for Reporting Results of Internet E-Surveys (CHERRIES). J. Med. Internet Res..

[19] Haesebaert F., Haesebaert J., Zante E., Franck N. (2020). Who Maintains Good Mental Health in a Locked-down Country? A French Nationwide Online Survey of 11,391 Participants. Health Place.

[20] Tennant R., Hiller L., Fishwick R., Platt S., Joseph S., Weich S., Parkinson J., Secker J., Stewart-Brown S. (2007). The Warwick-Edinburgh Mental Well-Being Scale (WEMWBS): Development and UK Validation. Health Qual. Life Outcomes.

[21] Trousselard M., Steiler D., Dutheil F., Claverie D., Canini F., Fenouillet F., Naughton G., Stewart-Brown S., Franck N. (2016). Validation of the Warwick-Edinburgh Mental Well-Being Scale (WEMWBS) in French Psychiatric and General Populations. Psychiatry Res..

[22] R Core Team (2020). R: A Language and Environment for Statistical Computing.

[23] Austin P.C. (2011). An Introduction to Propensity Score Methods for Reducing the Effects of Confounding in Observational Studies. Multivar. Behav. Res..

[24] Stranges S., Samaraweera P.C., Taggart F., Kandala N.-B., Stewart-Brown S. (2014). Major Health-Related Behaviours and Mental Well-Being in the General Population: The Health Survey for England. BMJ Open.

[25] Sedgwick P. (2013). Questionnaire Surveys: Sources of Bias. BMJ.

[26] Croutte P. (2020). Baromètre du Numérique 2019.

[27] Adelson J. (2019). Educational Research with Real-World Data: Reducing Selection Bias with Propensity Score Analysis. Pract. Assess. Res. Eval..

[28] Ginja S., Coad J., Bailey E., Kendall S., Goodenough T., Nightingale S., Smiddy J., Day C., Deave T., Lingam R. (2018). Associations between Social Support, Mental Wellbeing, Self-Efficacy and Technology Use in First-Time Antenatal Women: Data from the BaBBLeS Cohort Study. BMC Pregnancy Childbirth.

[29] Barone Gibbs B., Kline C.E., Huber K.A., Paley J.L., Perera S. (2021). COVID-19 Shelter-at-Home and Work, Lifestyle and Well-Being in Desk Workers. Occup. Med..

[30] Magee L.A., von Dadelszen P., Khalil A. (2021). COVID-19 and Preterm Birth. Lancet Glob. Health.

[31] Truong B.T., Lupattelli A., Kristensen P., Nordeng H. (2017). Sick Leave and Medication Use in Pregnancy: A European Web-Based Study. BMJ Open.

[32] Matvienko-Sikar K., Pope J., Cremin A., Carr H., Leitao S., Olander E.K., Meaney S. (2021). Differences in Levels of Stress, Social Support, Health Behaviours, and Stress-Reduction Strategies for Women Pregnant before and during the COVID-19 Pandemic, and Based on Phases of Pandemic Restrictions, in Ireland. Women Birth.

[33] Liu L., Xue P., Li S.X., Zhang J., Zhou J., Zhang W. (2020). Urban-Rural Disparities in Mental Health Problems Related to COVID-19 in China. Gen. Hosp. Psychiatry.

[34] Durankuş F., Aksu E. (2020). Effects of the COVID-19 Pandemic on Anxiety and Depressive Symptoms in Pregnant Women: A Preliminary Study. J. Matern.-Fetal Neonatal Med..

[35] Pedrosa A.L., Bitencourt L., Fróes A.C.F., Cazumbá M.L.B., Campos R.G.B., de Brito S.B.C.S., Simões E., Silva A.C. (2020). Emotional, Behavioral, and Psychological Impact of the COVID-19 Pandemic. Front. Psychol..

[36] (2021). Avril 2021: La Hausse de Mars Se Poursuit; Nombre de Naissances en 2021.

[37] Hagaman A., LeMasters K., Zivich P.N., Sikander S., Bates L.M., Bhalotra S., Chung E.O., Zaidi A., Maselko J. (2021). Longitudinal Effects of Perinatal Social Support on Maternal Depression: A Marginal Structural Modelling Approach. J. Epidemiol. Community Health.

[38] Aksoy Derya Y., Altiparmak S., AkÇa E., GÖkbulut N., Yilmaz A.N. (2021). Pregnancy and Birth Planning during COVID-19: The Effects of Tele-Education Offered to Pregnant Women on Prenatal Distress and Pregnancy-Related Anxiety. Midwifery.

[39] Niela-Vilén H., Auxier J., Ekholm E., Sarhaddi F., Asgari Mehrabadi M., Mahmoudzadeh A., Azimi I., Liljeberg P., Rahmani A.M., Axelin A. (2021). Pregnant Women’s Daily Patterns of Well-Being before and during the COVID-19 Pandemic in Finland: Longitudinal Monitoring through Smartwatch Technology. PLoS ONE.

[40] Coiro M.J., Asraf K., Tzischinsky O., Hadar-Shoval D., Tannous-Haddad L., Wolfson A.R. (2021). Sleep Quality and COVID-19-Related Stress in Relation to Mental Health Symptoms among Israeli and U.S. Adults. Sleep Health.

[41] Garbazza C., Hackethal S., Riccardi S., Cajochen C., Cicolin A., D’Agostino A., Cirignotta F., Manconi M. (2020). Polysomnographic Features of Pregnancy: A Systematic Review. Sleep Med. Rev..

[42] Lagadec N., Steinecker M., Kapassi A., Magnier A.M., Chastang J., Robert S., Gaouaou N., Ibanez G. (2018). Factors Influencing the Quality of Life of Pregnant Women: A Systematic Review. BMC Pregnancy Childbirth.

[43] Kempler L., Sharpe L.A., Marshall N.S., Bartlett D.J. (2020). A Brief Sleep Focused Psychoeducation Program for Sleep-Related Outcomes in New Mothers: A Randomized Controlled Trial. Sleep.

